# COVID-19 latent age-specific mortality in US states: a county-level spatio-temporal analysis with counterfactuals

**DOI:** 10.3389/fepid.2024.1403212

**Published:** 2024-11-11

**Authors:** Andrew B. Lawson, Yao Xin

**Affiliations:** ^1^ Department of Public Health Sciences, College of Medicine, Medical University of South Carolina, Charleston, SC, United States; ^2^ School of Medicine, Usher Institute, University of Edinburgh, Edinburgh, United Kingdom

**Keywords:** Bayesian, counterfactuals, spatio-temporal, COVID-19, mortality, age-specific

## Abstract

During the COVID-19 pandemic, which spanned much of 2020–2023 and beyond, daily case and death counts were recorded globally. In this study, we examined available mortality counts and associated case counts, with a focus on the estimation missing information related to age distributions. In this paper, we explored a model-based paradigm for generating age distributions of mortality counts in a spatio-temporal context. We pursued this aim by employing Bayesian spatio-temporal lagged dependence models for weekly mortality at the county level. We compared three US states at the county level: South Carolina (SC), Ohio, and New Jersey (NJ). Models were developed for mortality counts using Bayesian spatio-temporal constructs, incorporating both dependence on current and cumulative case counts and lagged dependence on previous deaths. Age dependence was predicted based on total deaths in proportion to population estimates. This latent age field was generated as counterfactuals and then compared to observed deaths within age groups. The optimal retrospective space–time models for weekly mortality counts were those with lagged dependence and a function of caseload. Added random effects were found to vary across states: Ohio favored a spatially correlated model, while SC and NJ favored a simpler formulation. The generation of age-specific latent fields was performed for SC only and compared to a 15-month, 13-county data set of observed >65 age population. It is possible to model spatio-temporal variations in mortality at the county level with lagged dependencies, spatial effects, and case dependencies. In addition, it is also possible to generate latent age-specific fields based on estimates of death risk (using population proportions or more sophisticated modeling approaches). More detailed data will be needed to make more calibrated comparisons for future epidemic monitoring. The proposed discrepancy tool could serve as a useful resource for public health planners in tailoring interventions during epidemic situations.

## Introduction

During the COVID-19 pandemic, which spanned much of 2020–2023 and beyond, daily case and death counts were recorded globally. Various hub sites provided access to these data streams.

Based on these data, various modeling exercises were conducted, mostly using national or state-level data. These modeling approaches predominantly focused on time series modeling of both case numbers and mortality (1–4). However, there are only limited examples of spatio-temporal modeling for case or death count data during the pandemic, despite clear evidence of spatial spread over time (5–9).

With the benefit of retrospectively stabilizing the count data generated during the pandemic, the CDC in the United States has collated a complete set of stabilized counts for cases and deaths, available on a weekly basis at the county level within states (https://data.cdc.gov/dataset/Weekly-United-States-COVID-19-Cases-and-Deaths-by-/yviw-z6j5). For the period from 22 January 2020 to 10 May 2023, data for a total of 173 weeks are available. Note that these 7-day spans are useful as they help eliminate random variations caused by reporting delays and misassignments. In this study, we examined available mortality and associated case counts, with a focus on estimating missing information related to age distributions. The aggregate count data from this source do not include age distributions. While age is usually recorded for individual cases, it is possible that these data are not available during certain outbreaks, even though the differential risk is age-related. For the CDC pandemic data, age distributions are not available in the weekly data county-level reports. In this paper, we compared a model-based paradigm for evaluating the optimal retrospective modeling of the space–time variation in mortality as a function of caseload. In addition, we explored the generation of age-specific distributions of mortality counts within a spatio-temporal context. To achieve this, we employed Bayesian spatio-temporal lagged dependence models for weekly mortality at the county level. Here, we present the results of an evaluation of age-specific data from South Carolina (SC), which is available to us, as a first step in comparing three US states at the county level: South Carolina, Ohio, and New Jersey (NJ). Our choice of data source is based on several criteria. First, we sought to consider a range of states with different population bases. South Carolina is a southern US state with a predominantly rural population, whereas New Jersey is a northern US state that is highly urbanized. Ohio, also a northern state, has a mix of urban and rural counties. The state population sizes in millions vary as follows: NJ, 9.3; Ohio, 11.8; and SC, 5.1. Second, it was apparent that non-pharmaceutical interventions (NPIs) were implemented differently in these states, leading to a heterogeneity in policy decisions and responses to the disease.

### Available data

We have access to a data set covering 173 weeks of county-level mortality and case counts of COVID-19.

**Figure 1:**
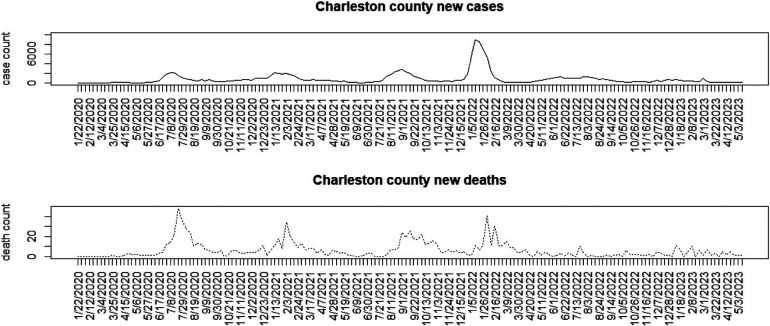
Charleston County, South Carolina: weekly case count and death count for the period from 22 January 2020 to 3 May 2023.

**Figure 2:**
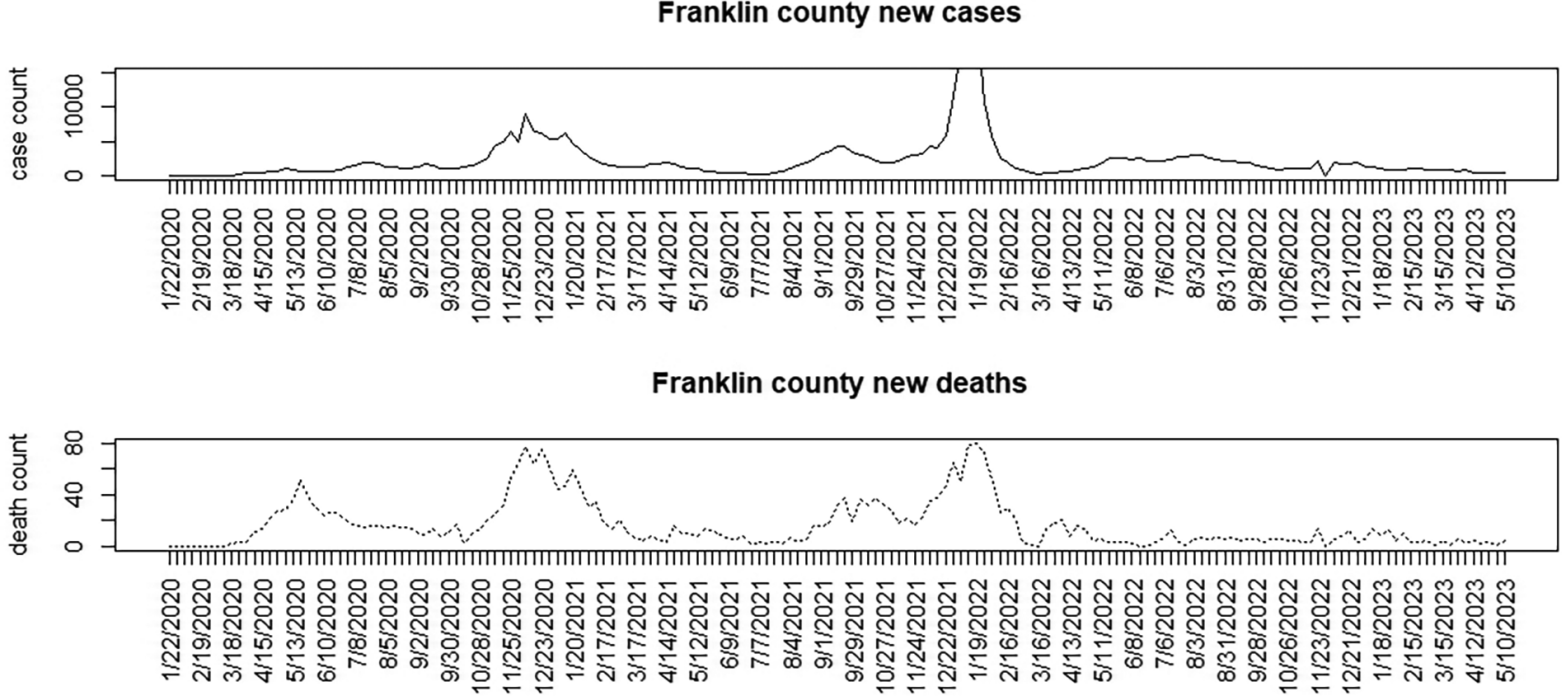
Franklin County, Ohio: weekly case count and death count for the period from 22 January 2020 to 3 May 2023.

**Figure 3:**
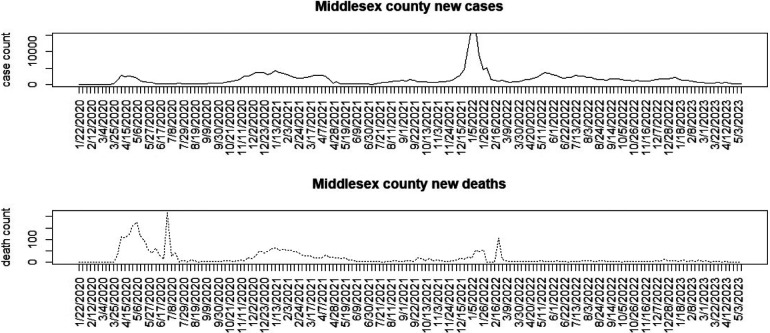
Middlesex County, New Jersey: weekly case count and death count for the period from 22 January 2020 to 3 May 2023.

**Figure 4:**
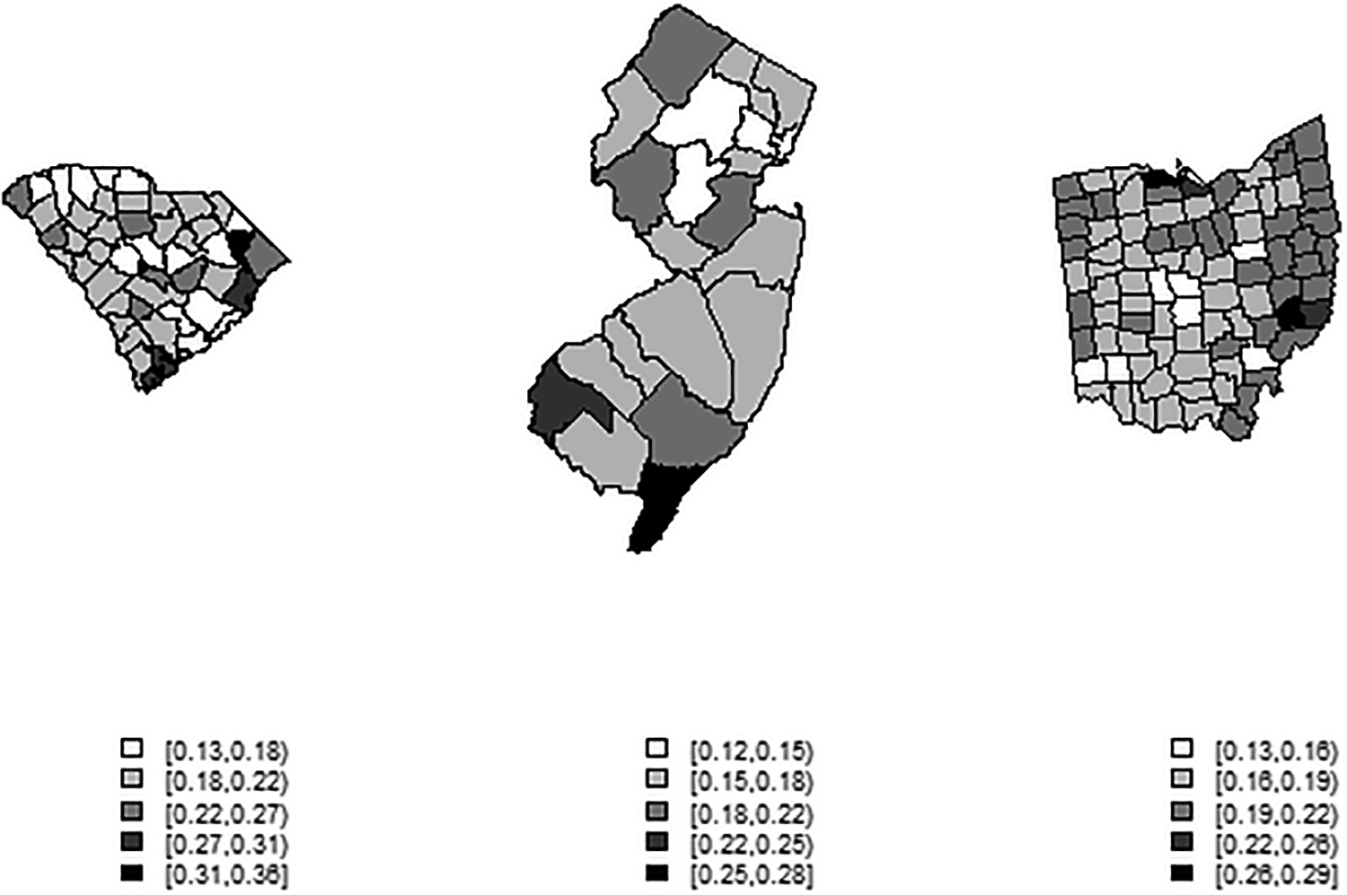
County-level maps of the proportion of population aged 65 and over in three US states (from left): South Carolina, New Jersey, Ohio (not to scale).

Figures 1–3 display the full 173-week period of case and death counts for selected counties in South Carolina, Ohio, and New Jersey. It is notable that, overall, the death time series follows the peaks and troughs of the case count series, with some variation. However, early mortality peaks in 2020 were observed in Ohio and New Jersey, but not in South Carolina. Figure 4 displays the proportion of population over 65 in three US states.

### Modeling strategy

Our modeling strategy consists of examining a range of relevant spatio-temporal mortality models to determine which are most effective for each state, followed by the imputation of the latent age fields across counties. The following quantities are available from the CDC Wonder site. For *m* counties within a state and *T* = 173 weeks,

$y_{i,j}^{d}$ and $y_{i,j}^{c}$ are the death count and case count for the *i*th county and *j*th week, respectively, $\mu_{ij}^{d}$ is the death rate, $\mu_{i,j}^{c}$ is the case rate, $y_{ij}^{dG65}$ is the death count in >65 age group in the *ij*th unit, and $\;y_{ij}^{dL65}$ is the death count in the <65 age group in the *ij*th unit.

### Aggregate models

As an overall aggregate mortality model, we used case and cumulative case dependencies and added a lagged mortality count term. A random-effect term, both spatially and temporally dependent, was also included (5, 6). We assumed a Poisson data model for the aggregate death count and modeled the mean level with a log link. Overdispersion was accounted for via a random effect at the unit level:

$$\begin{array}{rl} & y_{i,j}^{d}∼\text{Pois}(\mu_{i,j}^{d}),\\ & \text{log}(\mu_{ij}^{d})=\alpha_{0}+\alpha_{1}\text{log}(y_{ij}^{c})+\alpha_{2}\text{log}(y_{i,j-1}^{d})+\alpha_{3}\text{log}(T_{ij}^{y})+\zeta_{ij}, \end{array}$$

where $\mu_{ij}^{d}=\mu_{ij}^{dL65}+\mu_{ij}^{dG65}$.

Here, it is assumed that current case count (including an asymptomatic estimate, $y_{ij}^{c}$) and cumulative count ($T_{ij}^{y}$) are important, as is dependence on previous mortality count in the same county ($y_{i,j-1}^{d}$). This latter term introduces temporal lag dependence, alongside case dependence. The possibility that deaths could be lagged after case occurrence is accounted for by including the cumulative case count. This does not directly model the tapered lag in deaths but serves as a surrogate for that dependence. Finally, the random effect $\zeta_{ij}$ can be parameterized in various ways, depending on the context. Some variants of this general model have been considered. First, a base model is considered with only case dependence of the form:

(1) $$\text{log}(\mu_{ij}^{dL65}+\mu_{ij}^{dG65})=\alpha_{0}+\alpha_{1}\text{log}(y_{ij})+\alpha_{3}\text{log}(T_{ij}^{y})+\zeta_{i}\text{, }$$

where the random effect is spatial only with an uncorrelated prior distribution. This is termed an uncorrelated heterogeneity (UH) effect. This effect is assigned a prior distribution as

$\zeta_{i}=v_{i}∼N(0,\tau_{\varsigma }^{-1})$, with precision $\tau_{\varsigma }$ assigned a weakly informative gamma prior distribution, such as Ga (2,0.5).

As an extension to this base model, Equation 2,

(2) $$\text{log}(\mu_{ij}^{dL65}+\mu_{ij}^{dG65})=\alpha_{0}+\alpha_{1}\text{log}(y_{ij})+\alpha_{2}\text{log}(y_{i,j-1}^{d})+\alpha_{3}\text{log}(T_{ij}^{y})+\zeta_{i},$$

includes a lagged death dependence to account for temporal dependence on the mortality stream.

Equation 3 provides for an extra random component with a spatial dependence term added to Equation 1:

(3) $$\text{log}(\mu_{ij}^{dL65}+\mu_{ij}^{dG65})=\alpha_{0}+\alpha_{1}\text{log}(y_{ij})+\alpha_{3}\text{log}(T_{ij}^{y})+\zeta_{i},$$

where $\;\zeta_{i}=v_{i}+u_{i}$.

This represents a combination (or convolution) of spatial effects with $v_{i}∼N(0,\tau_{v}^{-1})$ as before and a spatial correlation term $u_{i}$. This term is assumed to have a conventional intrinsic conditional autoregressive prior distribution (ICAR), $u_{i}∼N({\bar{u}}_{\delta_{i}},\tau_{u}^{-1}/n_{\delta_{i}})$, which accounts for correlation via the neighborhood mean ${\bar{u}}_{\delta_{i}}$, with $\delta_{i}$ representing the neighborhood set (here defined for first-order neighbors of the *i*th region), and the number of neighbors as $n_{\delta_{i}}$ (10–12).

Finally, Equation 4 includes a lagged dependence term and also a convolution on the spatial random effects:

(4) $$\text{log}(\mu_{ij}^{dL65}+\mu_{ij}^{dG65})=\alpha_{0}+\alpha_{1}\text{log}(y_{ij})+\alpha_{2}\text{log}(y_{i,j-1}^{d})+\alpha_{3}\text{log}(T_{ij}^{y})+\zeta_{i},$$

where $\zeta_{i}=v_{i}+u_{i}$.

### Age-specific models

Note that for any model choice to estimate total deaths, we could assume a simple link between age groups or groups and total deaths. This disaggregation could take the following form for the >65 age group:

$yp_{i,j-1}^{dG65}∼Bin(\;p,y_{i,j-1}^{d})\text{, where}\;p=G65prop$. *G65prop* is the proportion of deaths in the >65 age group. As we did not observe the age-specific death counts, $yp_{i,j-1}^{dG65}$ remained unobserved and must be estimated. Of course, the $yp_{i,j-1}^{dG65}$ could be simulated from a binomial distribution based on the population proportion of >65 age group in a county (denoted $p^{G65}=G65prop$), so that

(5) $$yp_{i,j-1}^{dG65}∼Bin(p^{G65},y_{i,j-1}^{d}).$$

In the initial analyses of the mortality count data, it was found that different random-effect components were appropriate for the different states. Hence, variations in $\zeta_{ij}$ needed to be accounted for. However, to model dependence, as in our aggregate models, it is possible to propose the following approach for the age-specific variation:

(6) $$\begin{array}{rl} & y_{i,j}^{dG65}∼Pois(\mu_{i,j}^{dG65}),\\ & \text{log}(\mu_{i,j}^{dG65})=\text{log}(\;p^{G65})+\text{log}(\lambda_{i,j}^{dG65}),\\ & \text{log}(\lambda_{i,j}^{dG65})=\beta_{0}+\beta_{1}\text{log}(y_{i,j})+\beta_{2}\text{log}(T_{i,j}^{y})+\beta_{3}\text{log}(y_{i,j-1}^{dG65})+\zeta_{i,j}^{d}, \end{array}$$

where $\zeta_{i,j}^{d}=v_{i}\;\text{\textbackslash{},for\textbackslash{} SC\textbackslash{} and\textbackslash{} NJ\textbackslash{} and}\;\zeta_{i,j}^{d}=v_{i}+u_{i}\;\text{\textbackslash{},for\textbackslash{} OH}$.

The counts other than the specified age groups could be estimated by differencing, e.g., $yp_{i,j-1}^{dL65}=y_{i,j-1}^{d}-yp_{i,j-1}^{dG65}$.

This would be fitted jointly with the aggregate model for mortality:

$$\begin{array}{rl} & y_{i,j}^{d}∼Pois(\mu_{i,j}^{d}),\\ & \text{log}(\mu_{ij}^{d})=\alpha_{0}+\alpha_{1}\text{log}(y_{ij})+\alpha_{2}\text{log}(y_{i,j-1}^{d})+\alpha_{3}\text{log}(T_{ij}^{y})+\zeta_{ij},\\ & \mu_{ij}^{d}=\mu_{ij}^{dL65}+\mu_{ij}^{dG65}, \end{array}$$

where $\;\zeta_{ij}=v_{i}\;\text{\textbackslash{},for\textbackslash{} SC\textbackslash{} and\textbackslash{} NJ\textbackslash{} and\textbackslash{} }\zeta_{ij}=v_{i}+u_{i}\text{\textbackslash{} for\textbackslash{} OH}$.

Note that the $y_{i,j}^{dG65}$ would be latent and must be fitted jointly with the overall aggregate mortality model. As this involves extensive simulation of latent time-dependent fields, which is computationally demanding, the first approach adopted here was to generate estimated counts via simulation from the observed mortality totals within counties while simultaneously fitting the overall aggregate county-level time-dependent mortality models.

### Prior specification and sensitivity

In the models described above, we included different combinations of regression parameters. Conventional choices were made for the prior specification of these effects, generally opting for non-informative choices, although we also used weakly informative specifications (such as ICAR). For the regression parameters, we assumed zero mean Gaussian distributions: $\alpha_{*}∼N(0,\tau_{*}^{-1})$; for precision parameters, we assumed gamma prior distributions: $\tau_{*}∼Ga(a,b)$. We assumed that a=2.0 and b=0.5, which are weakly informative; these values are usually favored in Markov Chain Monte Carlo (MCMC) sampling, as they prevent sampling at an asymptote of 0. We also varied a,b specifications to assess prior sensitivity (a:1.0,0.5andb:0.1,0.2). We found that, while some posterior mean estimated regression parameters varied, the overall resulting model choice based on Watanabe–Akaike information criterion (WAIC) differences remained consistent. The resulting best-fit model was still identified. Hence, the inference appears robust to this type of variation (increased non-informativeness).

### Aggregate model fitting

To determine the best model for generating age-specific counts, we first examined the differences in overall goodness of fit using the WAIC measure. A choice of whether to use retrospective goodness of fit or predictive loss was considered (13). However, since we focused on developing a tool for the retrospective assessment of policy decisions, a retrospective fit measure was deemed most appropriate. Models were fitted using the MCMC posterior sampling R package Nimble. Convergence was checked using Geweke diagnostics with single chains. Models usually converged within 10,000 iterations, with a burn-in of 2,000. Models with ICAR components often required additional iterations to achieve convergence. We estimated the overall WAIC for each state when fitting the space–time-dependent models (models 1–4) described above. WAIC values are available for any fitted Nimble model. Interpretation of WAIC values follows the “small is better” criterion. Smaller values indicate a closer fit. A difference of around 3–5 indicates a significant difference in model ability. The code for selected Nimble models is available at https://github.com/AndrewBLawson/Age_structure/tree/CODE.

Table 1 displays the result of these fits for SC, NJ, and Ohio.

**Table 1:** WAIC values for different models using aggregate data.

	SC		NJ		OH	
	WAIC	pWAIC	WAIC	pWAIC	WAIC	pWAIC
Base model (1)	22,684.5	453.65	18,667.6	610.79	42,057.7	582.79
Lagged death dependence (2)	22,590.3^a^	600.83	17,205.0^a^	656.88	41,536.9	730.11
Spatial convolution (3)	22,667.9	444.97	18,673.6	586.38	41,530.5	724.25
Lagged plus spatial convolution (4)	22,600.6	598.87	17,247.1	669.46	41,519.1^a^	724.48

pWAIC, WAIC penalty.

^a^ Lowest WAIC for each area.

For the SC and NJ counties, the best descriptive model for deaths appears to be one with a lagged death dependence. However, for the Ohio counties, spatial models performed uniformly better, with the lowest WAIC model including lagged dependence and a spatial convolution term. It is worth noting that all models include an uncorrelated spatial heterogeneity term by default.

### Estimation of latent age-specific counts

To assess the extent to which age-specific estimates are related to observed counts, we approached the Departments of Health in each state. Although most US states maintain COVID-19 dashboards with case and mortality data, they typically do not provide county-level disaggregated age structures, which are required for these analyses. However, through a special request to the SC Department of Health and Environmental Control (SCDHEC), we obtained age-specific, county-level data for SC. These data span a limited time period during the pandemic and exhibit various missing data patterns at the county level. This missingness led us to consider a subset of data, both in terms of time and county inclusion. In fact, only 13 counties in SC have complete mortality counts: Aitken, Anderson, Beaufort, Charleston, Clarendon, Florence, Greenville, Horry, Kershaw, Lexington, Richland, Sumter, and York. The period of completeness is limited to 15 months, from March 2020 to May 2021. Hence, for comparative purposes, the only way to assess the estimated divergence between simulated counterfactual age-specific counts and observed counts is to aggregate the data by month and restrict the comparison to these 13 counties with complete data. Although the underlying mortality models can be fitted to the entire SC county set over 173 weeks, the comparison must be confined to the 13 counties and 15-month period.

Age-specific data requests for the NJ and OH are still pending, and the analysis of these age-specific data will be the subject of a subsequent publication. Hence, for initial evaluation, the focus of this paper is on the assessment of the available data from SC.

## Results

**Figure 5:**
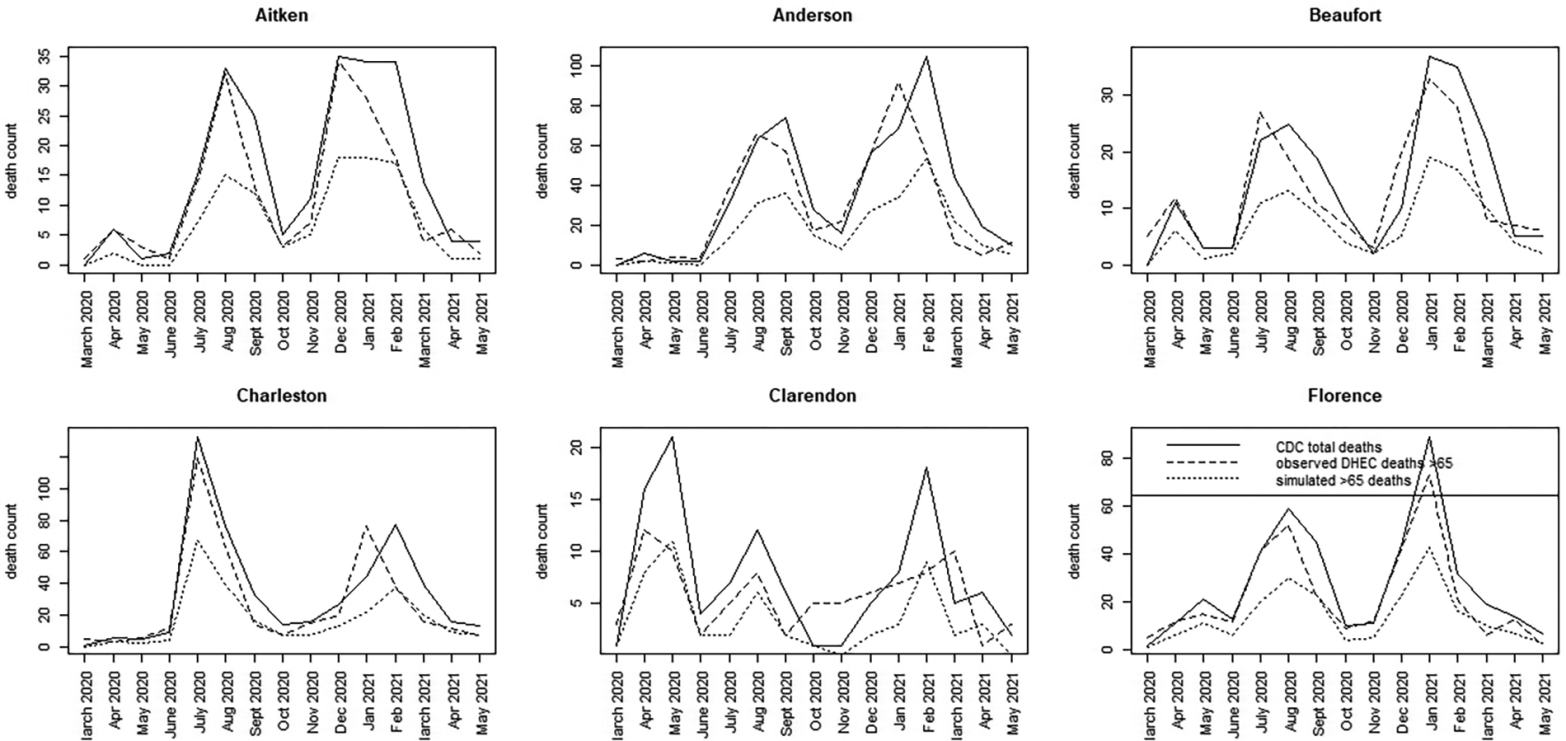
Time series plots for six counties in SC (Aitken to Florence) showing the CDC total death count, the SCDHEC-observed death count for >65 age group, and the counterfactual for the >65 age group.

**Figure 6:**
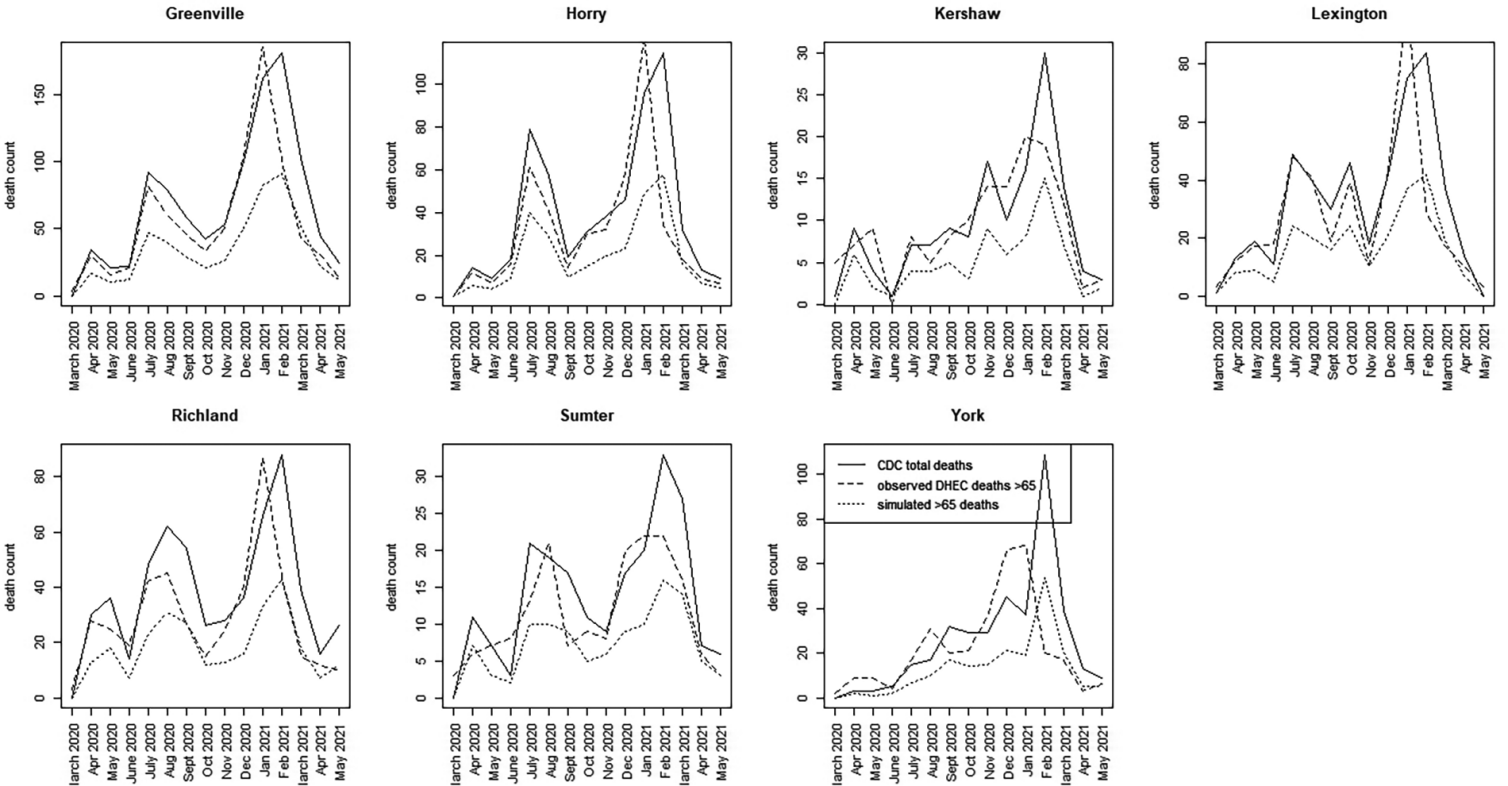
Time series plots for seven counties in SC (Greenville to York) showing the CDC total death count, the SCDHEC-observed death count for >65 age group, and the counterfactual for the >65 age group.

For the SC data, we have assumed a basic age-specific counterfactual model. Since we had access to a subset of county-level data, we assumed that the counterfactual population proportion is generated using a binomial model with $p^{G65}$, where the simplest model for the population proportion is employed. This model was fitted jointly with the best WAIC-based spatio-temporal model for total county-level mortality. Alternative specifications, such as variants of Equation 6, can be explored in future evaluations. Figures 5, 6 display time series plots of total deaths per county as recorded by CDC, the observed deaths in the >65 age group as recorded by SCDHEC by county, and the counterfactual deaths in the >65 age group generated from Equation 5. Note that the reduced time series of 15 months begins in March 2020 and ends in May 2021.

A number of features stand out in these data. First, the counterfactuals generated from the total counts track the total counts for each county as expected in proportion to the >65 age population. This suggests that the counterfactual model can act as a benchmark for measuring discrepancies in the actual death count, as demonstrated by the observed data. Second, there is considerable variation between counties in the form of the time series and the timing of mortality peaks. Charleston County demonstrates two large peaks centered around July 2020, with a smaller peak around February 2021. This suggests that suppression of severe infection may have been more successful there. In contrast, Greenville County demonstrates almost the reversed peaking pattern, with a small peak in July 2020 and a large peak around January and February 2021. In fact, many counties in SC show this lagged peaking pattern, with the majority of counties showing their largest peaks in January–February 2021.

For the most part, the SCDHEC data mirror the CDC-observed deaths in proportion, with some slight lag effects. However, the discrepancy between the observed count and the counterfactual estimates is notable in most counties.

**Figure 7:**
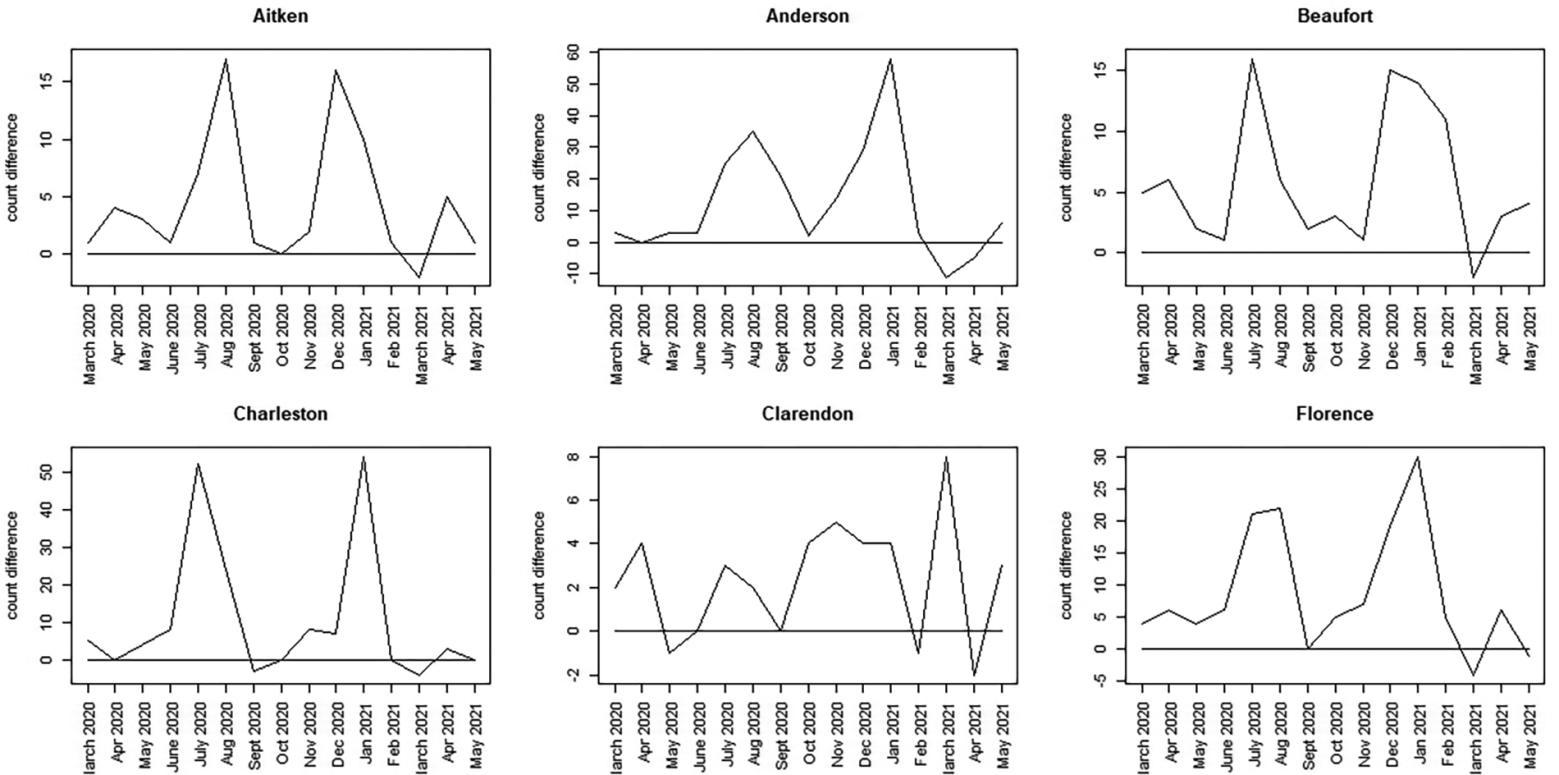
Discrepancy time series for six counties in SC (Aitken to Florence): difference computed as SCDHEC mortality count minus the counterfactual count generated from the CDC death count.

**Figure 8:**
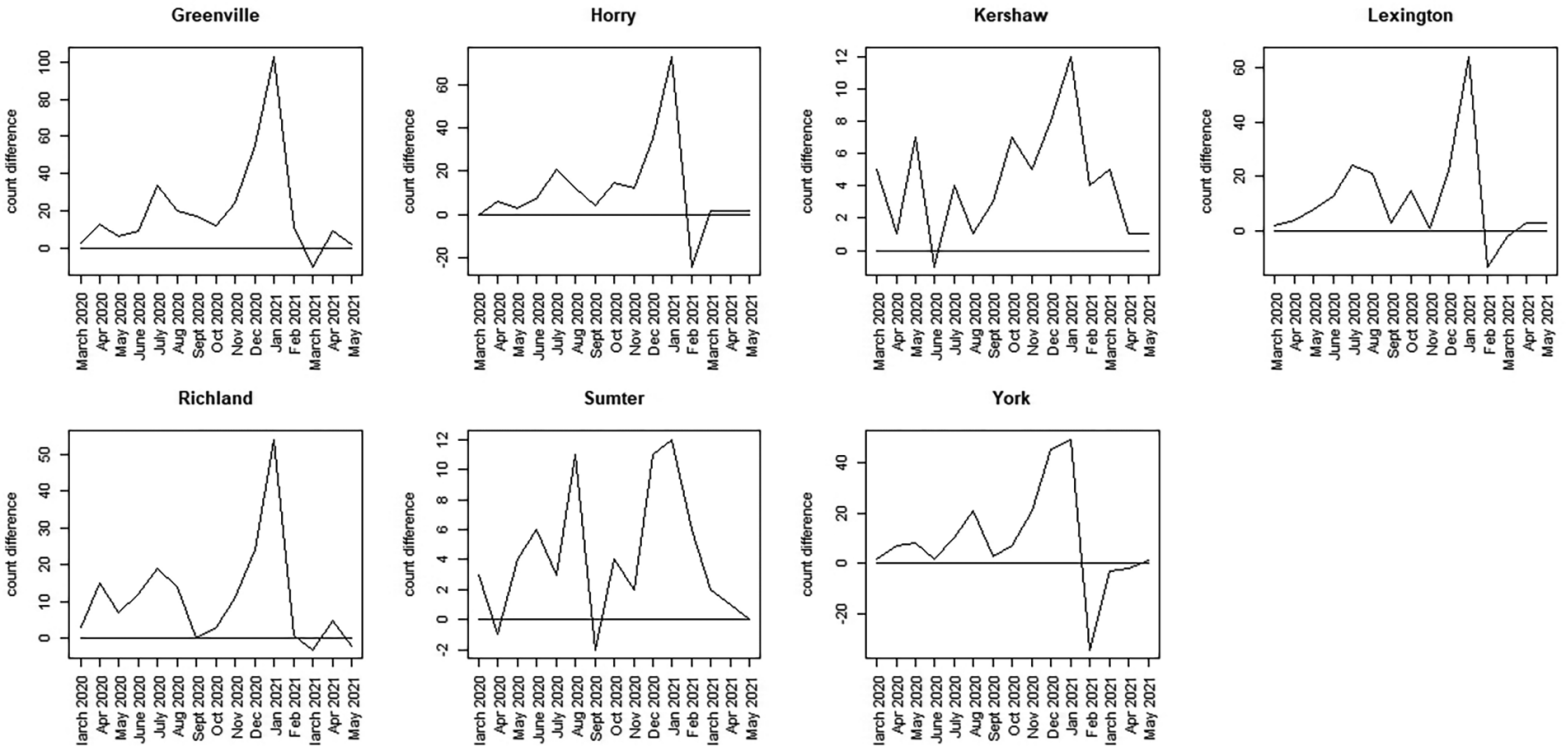
Discrepancy time series for seven counties in SC (Greenville to York): difference computed as SCDHEC mortality count minus the counterfactual count generated from the CDC death count.

To assess more clearly how the observed count differs from the counterfactual estimates, we calculated a time series discrepancy measure, reporting the unadjusted difference between observed and counterfactual data. Figures 7, 8 display this discrepancy measure over the 15-month period of focus.

The most notable feature of these plots is that, during the earliest part of the pandemic, the observed death count exceeds the counterfactual predicted from the population proportion in most counties.

However, the selected counties show lower discrepancies in the earlier period, with large spikes around winter 2020–2021 (Greenville, Horry, Richland, Lexington, York). In contrast, other counties display bivalued spikes in discrepancy around July 2020 and January 2021 (Charleston, Aitken, Anderson, Beaufort, Florence, Kershaw, Sumter). It should be noted, however, that the scale of the discrepancies varies between counties. In some cases, the discrepancy reaches a magnitude of 100 (Greenville), whereas in others, it is as small as 15 (Beaufort). By March 2021, in all cases, the discrepancy flips, with observed counts falling below the counterfactual estimates. This negative discrepancy is usually small in magnitude and appears to relate to the end of the winter wave in 2020–2021, just before the delta wave emerged in spring/summer 2021. The first delta variant case in the United States was identified in March 2021.

## Discussion

Overall, these results suggest significant differences in the county-level control of COVID-19 mortality within this state. A disproportionate mortality excess was experienced across most counties during the winter of 2020–2021, with Greenville showing the highest excess among the 13 counties sampled. It is also notable that, unlike other counties, Charleston was more successful in suppressing the second winter wave compared to other counties. Our unscaled discrepancy measure demonstrates these differences quite clearly and provides a basis for monitoring spatial and temporal variations in mortality within a state. This measure therefore can form the basis of a useful policy tool, helping to highlight differences in mortality trends. This could allow better design of intervention activities.

We conducted a sensitivity analysis related to prior specification to assess whether prior choice impacted the final model fitting results and the related derived measures. We considered variations in the parameterization of precision prior distributions but found little impact on the posterior parameter estimates.

Further refinements to the approach and the discrepancy measure could be made. First, population proportions could be replaced by death rates to account for specific mortality experiences, moving toward an excess death estimation. However, in novel pandemics, such rates are either unavailable or evolving with time, making them potentially unstable. Population proportions remain relatively static and could be assumed constant over time periods. A further extension, as discussed above, is the use of a more comprehensive age-specific model, which could differ from the parent model. It could easily be the case that the lagged dependency over time for a specific age group differs from that of the overall population, allowing for a more sensitive modeling approach. However, it should be kept in mind that age group is a latent field in this application, and any structure assumed must support the identifiable estimation of that field.

In addition, some changes or improvements could be made to the discrepancy measure. First, a confidence interval could be used for the counterfactual, either in addition to or instead of the posterior predictive mean, to provide a measure of extremity for the observed discrepancy. The observed count provided by SCDHEC was fixed. In addition, to facilitate comparisons, a proportionate discrepancy could be employed, which could scale the discrepancy based on the total mortality in each county.

NPIs were implemented during the pandemic at different times an for varying duration across different US states. So far, we have not included specific information about the nature of these interventions in this work. In the case of SC, which was used for our evaluation, the lockdown occurred only during April 2020 and was almost completely lifted by May of that year. Hence, the effect of an NPI was limited by the summer of 2020, when the first large wave occurred.

In future work, we would like to improve the data quality by incorporating CDC-based age data to provide a better testing resource. In addition, we would like to introduce the stringency index, which is compiled by the Oxford Covid-19 Government Response Tracker (OxCGRT: http://bsg.ox.ac.uk/covidtracker), which focuses on the stringency of NPIs during the pandemic. In addition, the viral variant proportion would serve as a useful time-varying additional confounder for the period in question. We have not yet examined vaccination coverage, as the roll-out did not take place until later in 2021.

## Conclusions

We have demonstrated that different spatio-temporal models apply optimally in different locales, with spatially correlated prior components (for example, ICAR) sometimes playing an important role in capturing the overall spatial variation over time. We also demonstrated that it is possible to generate counterfactuals for age-specific COVID-19 mortality rates and use them to assess the effectiveness of control measures in reducing fatalities during the pandemic. Aggregation of counts to month intervals and the restriction to only 13 counties is a major drawback of this study. Yet to be fully explored is extending the focus to Ohio and New Jersey with additional data requests.

Alternative mechanisms for generating age-specific counterfactuals could be explored. The first option is to use model-based generation, similar to the main overall count model forms, which involves generating latent fields in space–time with unobserved parameters. Another extension could be to use expected age-specific rates from a standard population (12), which would more closely reflect expected deaths. Our use of population-based proportionate rates provides a fast and simple, albeit crude, approach for generating age-specific counts.

While we utilized retrospective models and assessed their goodness of fit, it is possible to consider fixed data windows that evolve over time, allowing for adjustments to the optimal models to be used without changing the overall retrospective approach. Finally, our future work will consider extending the age-specific discrepancy measure using more sophisticated scoring rules (14, 15) and including NJ and OH in the evaluation.

## Data Availability

Publicly available datasets were analyzed in this study. This data can be found here: https://data.cdc.gov/dataset/Weekly-United-States-COVID-19-Cases-and-Deaths-by-/yviw-z6j5/data_preview.
